# Supplementary material for: Kinetochore- and chromosome-driven transition of microtubules into bundles promotes spindle assembly
Source: Nat Commun. 2022 Nov 27;13:7307. doi: 10.1038/s41467-022-34957-4 (PMC9701229; doi:10.1038/s41467-022-34957-4)
Supplement: Supplementary file 1 — Supplementary Information [file 41467_2022_34957_MOESM1_ESM.pdf]

Kinetochore- and chromosome-driven transition of microtubules into bundles promotes spindle assembly

Supplementary Information

Jurica Matković[1], Subhadip Ghosh[2], Mateja Ćosić[1], Susana Eibes[3], Marin Barišić[3,4], Nenad Pavin[2], Iva M. Tolić[1*]

[1]Division of Molecular Biology, Ruđer Bošković Institute, 10000 Zagreb, Croatia
[2]Department of Physics, Faculty of Science, University of Zagreb, 10000 Zagreb, Croatia
[3]Cell Division and Cytoskeleton, Danish Cancer Society Research Center, 2100 Copenhagen, Denmark
[4]Department of Cellular and Molecular Medicine, Faculty of Health Sciences, University of Copenhagen, 2200 Copenhagen, Denmark

*Correspondence to: tolic@irb.hr

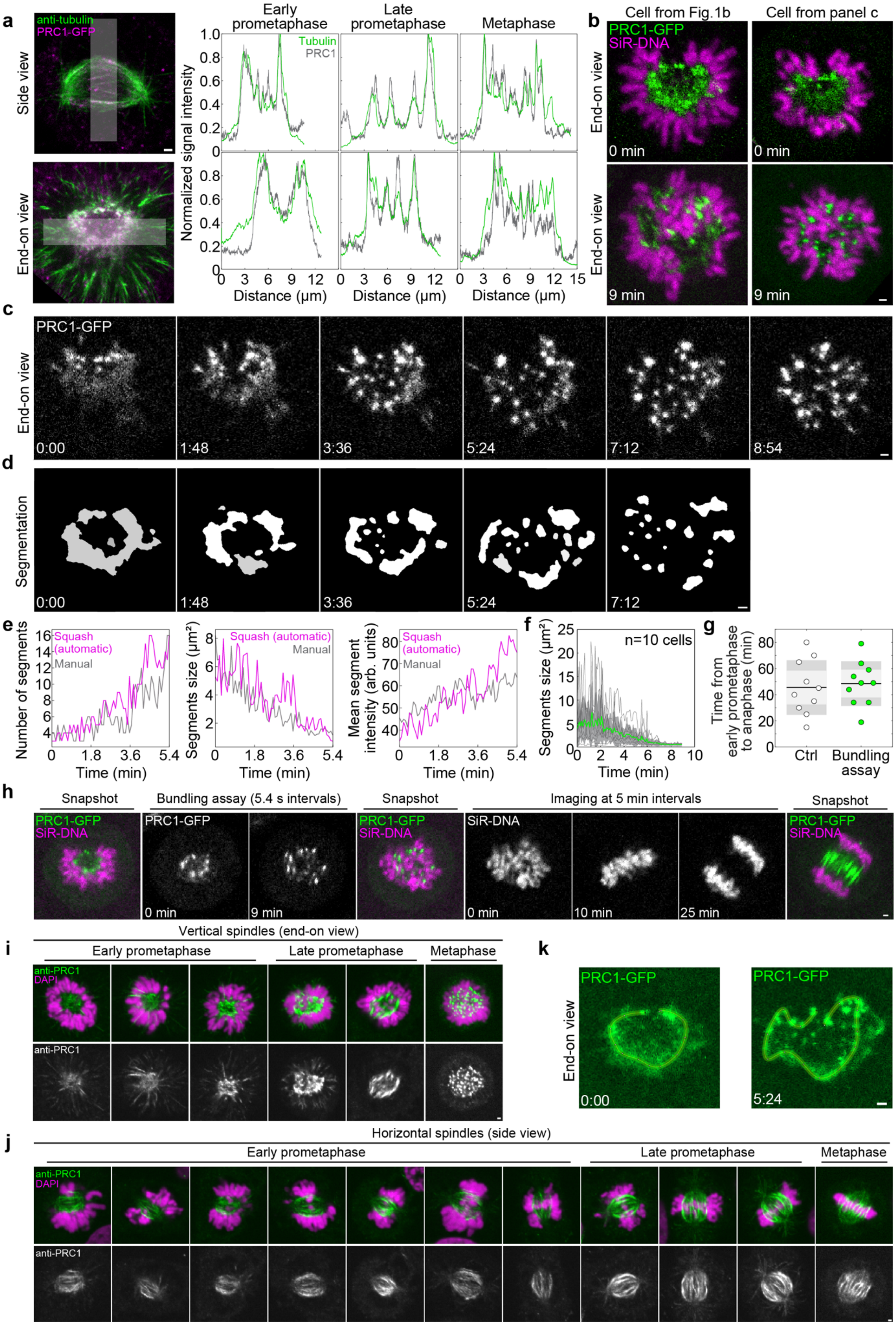

a
Side view
anti-tubulin
PRC1-GFP
End-on view
Early prometaphase
Late prometaphase
Metaphase
Tubulin
PRC1
Normalized signal intensity
Distance (µm)
b
Cell from Fig.1b
Cell from panel c
PRC1-GFP
SiR-DNA
0 min
9 min
c
PRC1-GFP
0:00
1:48
3:36
5:24
7:12
8:54
d
Segmentation
e
Squash (automatic)
Manual
Number of segments
Segments size (µm²)
Mean segment intensity (arb. units)
Time (min)
f
n=10 cells
g
Time from early prometaphase to anaphase (min)
Ctrl
Bundling assay
h
Snapshot
Bundling assay (5.4 s intervals)
Imaging at 5 min intervals
10 min
25 min
i
Vertical spindles (end-on view)
anti-PRC1
DAPI
j
Horizontal spindles (side view)
k
5:24

Supplementary Fig. 1: Additional characterization of the network-to-bundles transition of PRC1-crosslinked microtubules.

a, STED images of tubulin (green) and confocal images of PRC1 (magenta) from Fig. 1a, with superimposed lines drawn in Fiji (left) used to measure line intensity profiles on horizontally oriented spindles (top), and vertically oriented spindles (bottom) in different phases as indicated (right). Both PRC1-GFP and anti-tubulin intensity profiles are shown. Intensity is normalized to the maximum value. Note the colocalization of the PRC1 and tubulin signal. Similar observations were made in more than 10 spindles from 3 independent experiments. **b**, Midplane of a vertically oriented spindles in a HeLa-Kyoto BAC cells stably expressing PRC1-GFP (green) with DNA labeled by SiR-DNA (magenta), before (top) and after (bottom) antiparallel bundle formation (9 minutes). **c**, Time lapse images (single plane) of a cross-section of a vertically oriented prometaphase spindle in a HeLa-Kyoto BAC cell stably expressing PRC1-GFP, starting with the prometaphase rosette. **d**, Squassh segmentation for time-lapse images of PRC1-GFP in the midplane of a vertically oriented prometaphase spindle from Fig. 1b. **e**, Validation of the squassh segmentation method by manual measurements on a representative spindle. Graphs show the number of segments, their size and mean intensity (from left to right). In **b**-**d**, similar observations were made in more than 10 spindles from 5 independent experiments. **f**, Mean size of PRC1 segments in the midplane over time in HeLa-Kyoto BAC cells stably expressing PRC1-GFP, obtained using squassh segmentation. Gray lines represent individual cells, green line the mean and gray areas the standard deviation (n=10 cells in 10 independent experiments). **g**, Time from early prometaphase to anaphase in control cells, which were imaged "gently" at 5-minute intervals (left, 'Ctrl', mean±s.e.m.= 45.5±6.6 min, n=10), and in cells imaged as in the bundling assay and subsequently at 5-minute intervals (right, 'Bundling assay', 48.5±5.4 min, n=10; an example is shown in panel **h**; note that 11 cells were imaged in total, but one was still in metaphase at the end of imaging at 90 min). In both groups HeLa-Kyoto BAC cells stably expressing PRC1-GFP were used. The black line shows the mean; the light and dark grey areas mark 95% confidence interval on the mean and standard deviation, respectively. **h**, Images of a single HeLa-Kyoto BAC cell stably expressing PRC1-GFP (green or white) and stained with SiR-DNA (magenta or white, as indicated), which was subjected to the bundling assay and tested for anaphase entry. From left to right: snapshot showing DNA and PRC1 typical for vertically oriented spindles in early prometaphase; the first and the last image from the bundling assay; snapshot after the bundling assay showing that the cell is in late prometaphase; selected images from time-lapse imaging of DNA at 5-minute intervals until anaphase; snapshot showing DNA and PRC1 signal typical for anaphase. This image sequence demonstrates that the cell subjected to the bundling assay subsequently entered anaphase. **i** and **j**, A gallery of vertically oriented spindles (**i**) and horizontally oriented spindles (**j**) from untransfected HeLa cells immunostained for PRC1 (green in the top row, white in the bottom row) and stained with DAPI to show DNA (magenta). The spindles are ordered from left to right according to phases, demonstrating a network-to-bundles transition of PRC1 in cells that were neither transfected with PRC1-GFP nor subjected to the intense imaging protocol of the bundling assay. Similar observations were made in more than 10 spindles from 5 independent experiments. **k**, Lines drawn in Fiji along the periphery of the spindle midplane, where the PRC1 network is located in prometaphase, which were used to measure line intensity profiles in Fig. 1e,f. All scale bars, 1 μm.

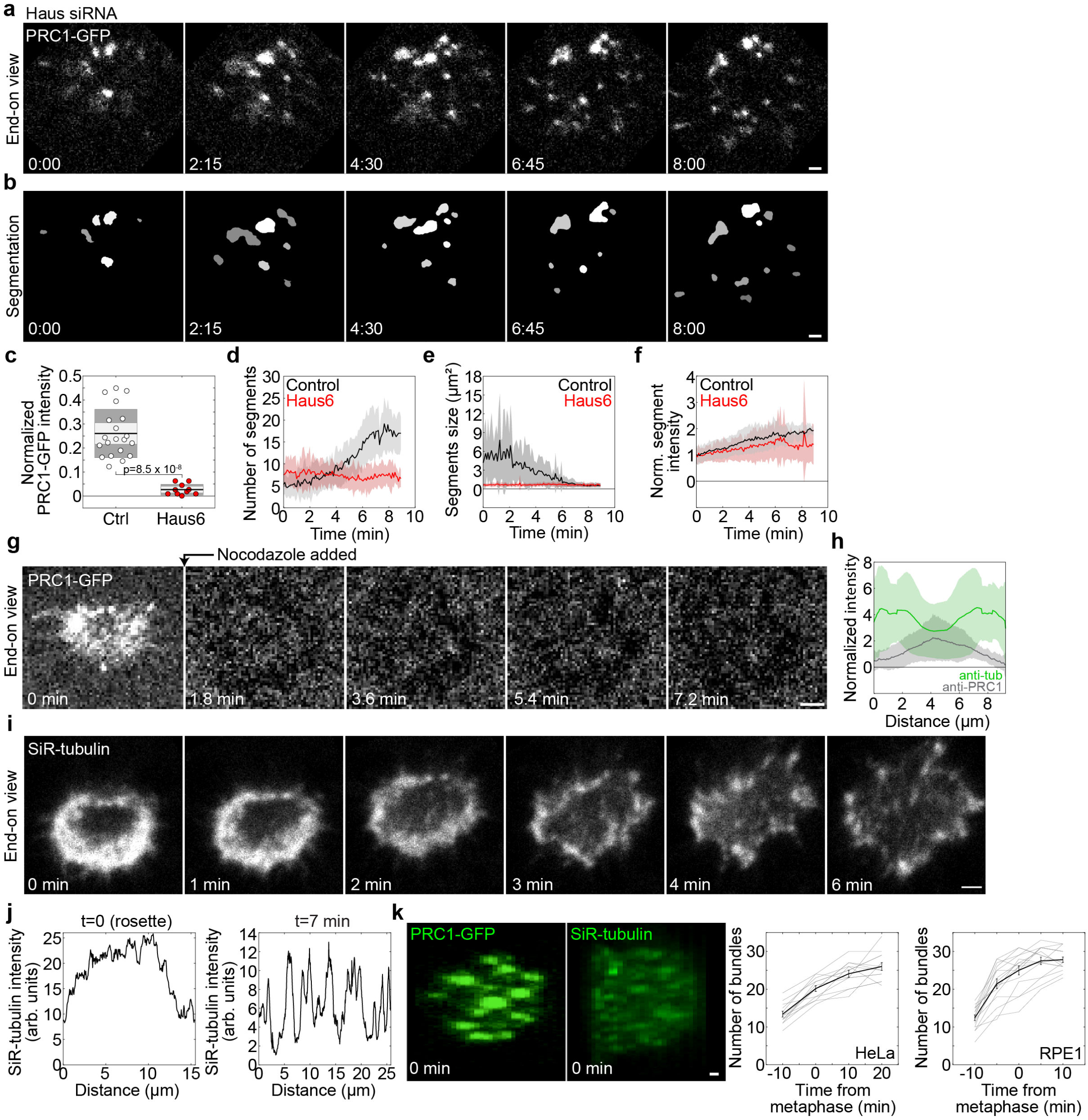

Supplementary Fig. 2: Additional characterization of PRC1 and tubulin during bundle formation.

a, Time lapse images of the midplane (single plane) of a vertically oriented prometaphase spindle in a Haus6-depleted HeLa-Kyoto BAC cell stably expressing PRC1-GFP, starting with the prometaphase rosette. Time is shown in minutes:seconds. **b**, Squassh segmentation results of the images shown in **a**. In **a**-**b**, similar observations were made in 10 and 9 spindles, respectively, from more than 3 independent experiments. **c**, Mean intensity of PRC1-GFP in a region on the opposite side of the bundles that lack chromosomes in the early prometaphase network in control (n=20) and Haus6-depleted (n=10) HeLa cells expressing PRC1-GFP. PRC1-GFP intensity is normalized to the cytoplasmatic PRC1-GFP intensity. Black line shows the mean; the light and dark grey areas mark 95% confidence interval on the mean and standard deviation, respectively. **d**, Number of PRC1 segments in the midplane over time in Haus6-

depleted (red) and control (black, gray) HeLa-Kyoto BAC cells stably expressing PRC1-GFP (n=10 cells for control and 9 cells for Haus6 depletion), obtained using squassh segmentation. **e**, Segment size and **f**, normalized segment intensity of PRC1-GFP segments from the same cells as in **d**. **g**, Time lapse images of the midplane (single plane) of a vertically oriented prometaphase spindle in a HeLa-Kyoto BAC cell stably expressing PRC1-GFP, starting with the prometaphase rosette. Time when nocodazole was added is indicated. Similar observations were made in more than 10 spindles from 3 independent experiments. **h**, Intensity line plots of the anti-PRC1 (gray) and anti-tubulin (green) signal along the pole-to-pole axis in horizontally oriented early prometaphase spindles (rosette stage) of non-transfected immunostained HeLa cells, n=20 cells from 2 independent immunostainings. Intensity was normalized to the cytoplasmatic anti-PRC1 and anti-tubulin intensity, respectively. Note that the PRC1 intensity is highest in the middle of the spindle, whereas tubulin intensity is highest away from the middle, i.e., close to the poles. **i**, Time lapse images of the midplane (single plane) of a vertically oriented prometaphase spindle in a HeLa-Kyoto BAC cell stably expressing PRC1-GFP and stained with SiR-tubulin, starting with the prometaphase rosette. Only SiR-tubulin channel was imaged. **j**, SiR-tubulin intensity line plots along the periphery of the spindle midplane at t=0 and t=7 minutes from the cell shown in **i**. In **i**-**j**, similar observations were made in more than 3 spindles from 3 independent experiments. **k**, Reconstructed (rotated by 90 degrees) images of the midplane of a horizontally oriented spindle in a HeLa-Kyoto BAC cell stably expressing PRC1-GFP (left image) and in an hTERT-RPE1 cell expressing CENP-A-GFP and centrin1-GFP labeled with SiR tubulin (right image). Graphs show the number of bundles over time for the HeLa cell line (left, n=13 cells) and the RPE1 cell line (right, n=16 cells), corresponding to the images on the left. Black line (mean) with error bars (s.e.m.), together with gray lines representing individual cells are shown; time zero is the beginning of metaphase. In **d**-**f** and **h,** colored surfaces around the central lines (mean) represent standard deviation. Images were acquired from at least 3 independent experiments for each cell line. All scale bars, 1 μm.

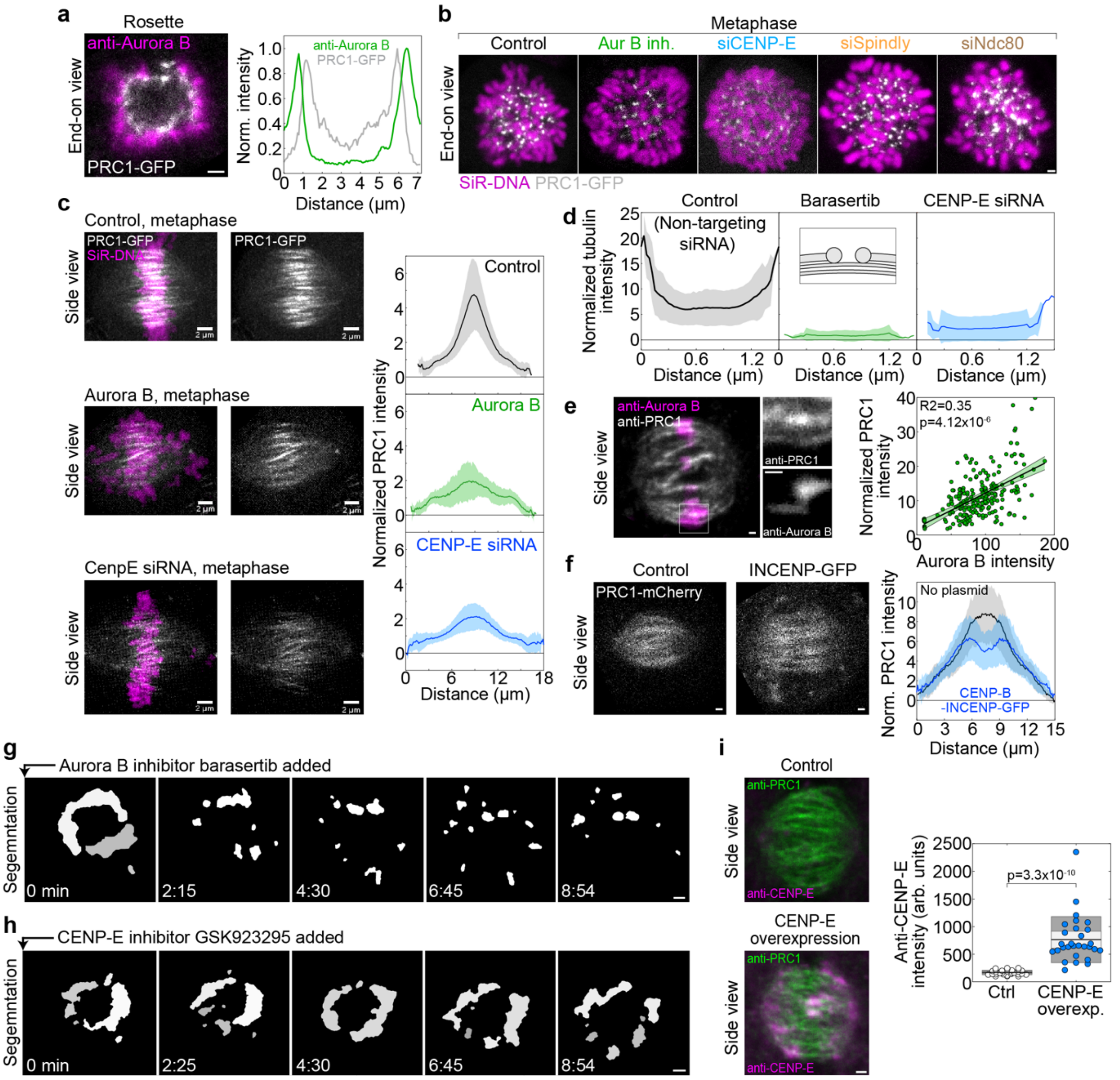

Supplementary Fig. 3: Additional characterization of the role of kinetochores in overlap bundle formation.

a, Left, image of a vertically oriented prometaphase rosette immunostained for Aurora B (magenta) in a HeLa-Kyoto BAC cell stably expressing PRC1-GFP (white). Right, intensity line plot of PRC1-GFP (grey) and anti-Aurora B (green) along a line drawn across the spindle cross-section, passing through the center. Line thickness was 1/3 of the spindle cross-section. Intensity is normalized to the maximum value for each channel. Similar observations were made in more than 10 spindles from 3 independent experiments. **b**, Images of vertically oriented metaphase spindles in a HeLa-Kyoto BAC cell line stably expressing PRC1-GFP (white) from Fig. 2e. Chromosomes are labeled with SiR-DNA (magenta) to show the metaphase plate. Similar observations were made in more than 10 spindles from 3 independent experiments for every condition. **c**, Live-cell images of horizontally oriented metaphase spindles in HeLa-Kyoto BAC cells stably expressing PRC1-GFP (white) and stained with SiR-DNA (magenta). Control (top), Aurora B inhibition (middle) and CENP-E depletion (bottom),

maximum intensity of 41 planes. The corresponding intensity line profiles of PRC1-GFP are shown to the right, mean (central line) and standard deviation (colored surface). The line profiles were measured along a line drawn from pole to pole. Line thickness was 1/3 of the spindle width. Intensity was normalized to the cytoplasmatic PRC1 intensity. Scale bars, 2 μm. Number of cells: 56 for control, 32 for Aurora B inhibition, 43 for CENP-E siRNA. All measurements are from 3 independent experiments. **d**, Intensity line profiles corresponding to anti-tubulin signal in the bridging fiber; mean (central line) and standard deviation (colored surface); n=22 bundles from 14 cells for control, 11 bundles from 9 cells for Aurora B inhibition, and 10 bundles from 7 cells for CENP-E siRNA, from 3 independent immunostainings. Intensity was normalized to the cytoplasmatic anti-tubulin intensity. **e**, Image of a spindle immunostained for Aurora B (magenta) and PRC1 (white) in an untransfected HeLa cell in metaphase, sum intensity of 5 planes. Enlargements of anti-PRC1 and anti-Aurora B (both in white) within the square marked on the main image are shown to the right. Graph shows correlation between anti-PRC1 and anti-Aurora B signal on the corresponding centromere, n=404 PRC1-Aurora B pairs from 26 cells. Intensity was normalized to the cytoplasmatic anti-PRC1 and anti-Aurora B intensity, respectively. Linear regression is used. **f**, Live cell images of metaphase spindles in HeLa cells transfected with PRC1-mCherry (white) in a control cell (left image) and a cell transfected with CENP-B-INCENP-GFP (right image), maximum intensity of 41 planes. Graph shows intensity line plots of PRC1-mCherry along the spindle main axis, mean (central line) and standard deviation (colored surfaces), n=29 cells for control and n=28 for CENP-B-INCENP-GFP, 3 independent experiments per condition. Intensity was normalized to the cytoplasmatic PRC1 intensity. **g**, Squassh segmentation results for time-lapse images of the midplane of a Barasertib-treated vertically oriented prometaphase spindle in a HeLa-Kyoto BAC cell stably expressing PRC1-GFP, corresponding to Fig. 2j. **h**, Squassh segmentation results for cells treated with the CENP-E inhibitor GSK923295, corresponding to Fig. 3f. In **g**-**h**, similar observations were made in more than 10 spindles from 3 independent experiments. **i**, Images of horizontally oriented spindles in metaphase, immunostained for PRC1 (green) and CENP-E (magenta), in a control HeLa cell (top image) and after CENP-E overexpression (bottom image). Graph shows total CENP-E intensity on the spindle in control cells and after CENP-E overexpression, n=29 cells in each condition from 3 independent experiments. P-value from a two-tailed t-test is given. The black line shows the mean; the light and dark grey areas mark 95% confidence interval on the mean and standard deviation, respectively. Note that the overexpression level is roughly 5-fold. In all controls for siRNA experiments the cells were transfected with nontargeting siRNA. In **c**, **d** and **f**, colored surfaces around the central lines (mean) represent standard deviation. All scale bars except in **c**, 1 μm.

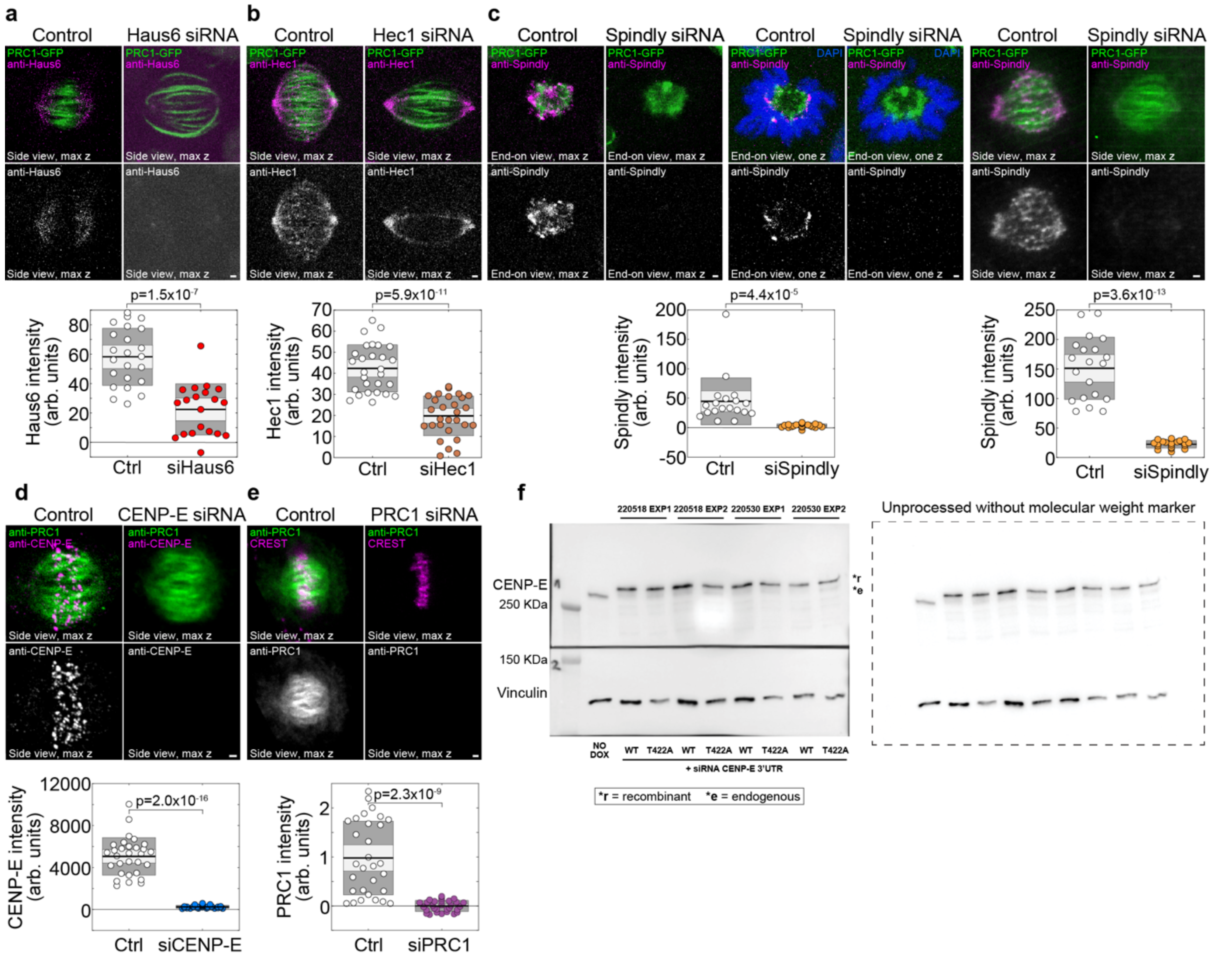

Supplementary Fig. 4: Quantification of protein depletion.
In each panel the images show control and depleted spindles, as indicated, with merged channels in the first row and only the channel of the depleted protein (white) in the second row. The graph under the images shows the quantification of the total signal intensity of the indicated protein on the spindle in control and depleted cells. For Hec1 depletion and the corresponding control, the total intensity of anti-Hec1 signal was measured in a region encompassing all DNA, i.e., excluding the poles. In **a**-**c**, HeLa-Kyoto BAC cell line stably expressing PRC1-GFP was used, whereas in **d**,**e**, untransfected HeLa cells were used. The number of cells was: (**a**) control, n=23, and Haus6 depletion, n=20, (**b**) control, n=28, and Hec1 depletion, n=28, (**c**) control, n=19, and Spindly depletion, n=20 for vertical spindles; control, n=20, and Spindly depletion, n=20 for horizontal spindles, (**d**) and (**e**), n=30 for each condition. Mean silencing was: (**a**) 62%, (**b**) 53%, (**c**) 93% (vertical spindles) and 85% (horizontal spindles), (**d**) 94%, (**e**) ~100% when the background signal is subtracted (data shown in panel **e**) and 86% without background subtraction (data not shown). All spindles were horizontally oriented (labeled 'side view'), in metaphase, and maximum-intensity projections are shown (labeled 'max z'), except in (**c**), where the left block shows maximum-intensity projections ('max z') of vertically oriented spindles ('end-on view'), the middle block shows the central planes ('one z') of the same spindles from the left block including DNA staining (DAPI) to show that the spindle is in early prometaphase, and the right block shows horizontally oriented

spindles (‘side view’) in prometaphase. Note that the Spindly spots are found at the edge of the central plane of the vertical control spindle (‘end-on view, one z’), marking kinetochores, and at kinetochores and spindle poles in the horizontal control spindle (‘side view’). The Spindly intensity graph on the left corresponds to the vertical spindles, and the graph on the right to the horizontal spindles. In all control experiments the cells were transfected with nontargeting siRNA. In the images in **a**-**e**, scale bars represent 1 μm, and similar observations were made in more than 10 cells from 3 independent experiments per condition. In the graphs in **a**-**e**, the black line shows the mean; the light and dark grey areas mark 95% confidence interval on the mean and standard deviation, respectively, and p-values from a two-tailed t-test are given. **f**, Western blot of cell lysate for CENP-E on doxycycline-treated cells expressing CENP-E wild-type (WT) and the T422A mutant. “NO DOX” signifies no doxycycline. Endogenous CENP-E was silenced using siRNA for CENP-E 3’UTR, as indicated. Stars mark the bands of recombinant (*r) and endogenous (*e) CENP-E. Four independent experiments are shown, and labels above the image on the left denote individual experiments.

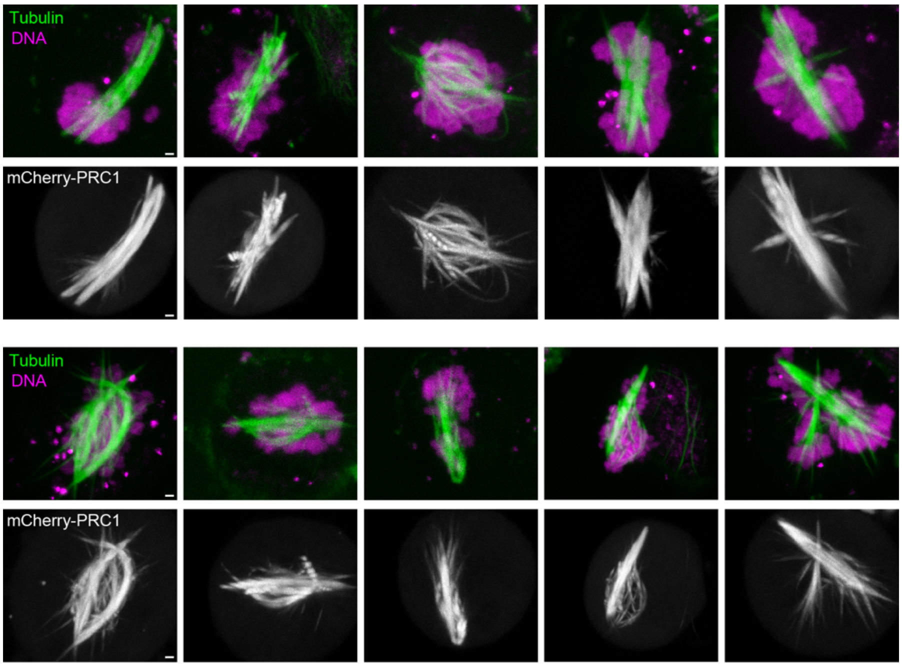

Supplementary Fig. 5: Additional images of spindles in cells with overexpressed PRC1. Examples of 10 spindles with overexpressed PRC1 in HeLa-TDS cells. Merged images show microtubules (SiR-tubulin, green) and DNA (Hoechst 33342, magenta), and the corresponding single-channel images below the merged ones show PRC1-mCherry (white). Similar observations were made in more than 10 cells from 3 independent experiments. Scale bars, 1 μm.
