# Supplementary material for: Kinetochore- and chromosome-driven transition of microtubules into bundles promotes spindle assembly
Source: Nat Commun. 2022 Nov 27;13:7307. doi: 10.1038/s41467-022-34957-4 (PMC9701229; doi:10.1038/s41467-022-34957-4)
Supplement: Supplementary file 2 — Description of Additional Supplementary Files [file 41467_2022_34957_MOESM2_ESM.pdf]

Description of Additional Supplementary Information

Supplementary Movie 1. Overlap bundle formation during early prometaphase.
Time lapse of a midplane of two vertically oriented prometaphase spindles (from Fig. 1b, left and S1c, right) in a HeLa-Kyoto BAC cell stably expressing PRC1-GFP starting with prometaphase rosette. A single plane is shown. Time interval between acquired images is 5.4 seconds. Time is shown in minutes:seconds. Scale bar, 1 µm.

Supplementary Movie 2. Bundle formation after Haus6 (augmin subunit) depletion.
Time lapse of a midplane of a vertically oriented prometaphase spindle in a Haus6-depleted HeLa-Kyoto BAC cell stably expressing PRC1-GFP starting with prometaphase rosette. A single plane is shown. Time interval between acquired images is 5.4 seconds. Time is shown in minutes:seconds. Scale bar, 1 µm.

Supplementary Movie 3. Bundle formation after Aurora B inhibition.
Time lapse of a midplane of a vertically oriented prometaphase spindle in a HeLa-Kyoto BAC cell stably expressing PRC1-GFP starting with prometaphase rosette. Barasertib was added just before the beginning of the movie. A single plane is shown. Time interval between acquired images is 5.4 seconds. Time is shown in minutes:seconds. Scale bar, 1 µm.

Supplementary Movie 4. Bundle formation after CENP-E inhibition.
Time lapse of a midplane of a vertically oriented prometaphase spindle in a HeLa-Kyoto BAC cell stably expressing PRC1-GFP starting with prometaphase rosette. GSK923295 was added just before the beginning of the movie. A single plane is shown. Time interval between acquired images is 5.4 seconds. Time is shown in minutes:seconds. Scale bar, 1 µm.

Supplementary Movie 5. Movement of overlap bundles during prometaphase.
Left, time lapse of a midplane of a vertically oriented prometaphase spindle in a HeLa-Kyoto BAC cell stably expressing PRC1-GFP starting with prometaphase rosette. The images were translated so that the position of the two bright bundles that are initially found in the upper region of the spindle is roughly constant. A single plane is shown. Time interval between acquired images is 2.7 seconds. Right, trajectories of the bundles from the movie on the left. Time is shown in minutes:seconds. Scale bar, 1 µm.

Supplementary Video 6. Chromosome congression to the spindle midplane causes spindle widening, end-on view.
Time lapse of the central part of a vertically oriented prometaphase spindle in a HeLa-Kyoto BAC cell stably expressing PRC1-GFP (green) starting with prometaphase rosette. DNA is stained with SiR-DNA (magenta). Maximum projection of 3 middle planes is shown. Time interval between acquired images is 30 seconds. Time is shown in minutes:seconds. Scale bar, 1 µm.

Supplementary Movie 7. Chromosome congression to the spindle midplane causes spindle widening, side view.
Time lapse of a horizontally oriented prometaphase spindle in a HeLa-Kyoto BAC cell stably expressing PRC1-GFP (green) starting with prometaphase rosette. DNA is stained with SiR-DNA (magenta). Sum of 41 planes is shown. Time interval between acquired images is 30 seconds. Time is shown in minutes:seconds. Scale bar, 1 µm.
