# Supplementary material for: Kinetochore- and chromosome-driven transition of microtubules into bundles promotes spindle assembly
Source: Nat Commun. 2022 Nov 27;13:7307. doi: 10.1038/s41467-022-34957-4 (PMC9701229; doi:10.1038/s41467-022-34957-4)
Supplement: Supplementary file 10 — Reporting Summary [file 41467_2022_34957_MOESM10_ESM.pdf]

nature portfolio

Corresponding author(s): Iva M. Tolic

Last updated by author(s): Nov 4, 2022

Reporting Summary

Nature Portfolio wishes to improve the reproducibility of the work that we publish. This form provides structure for consistency and transparency in reporting. For further information on Nature Portfolio policies, see our Editorial Policies and the Editorial Policy Checklist.

Statistics

For all statistical analyses, confirm that the following items are present in the figure legend, table legend, main text, or Methods section.

n/a	Confirmed	
☐	☒	The exact sample size (*n*) for each experimental group/condition, given as a discrete number and unit of measurement
☐	☒	A statement on whether measurements were taken from distinct samples or whether the same sample was measured repeatedly
☐	☒	The statistical test(s) used AND whether they are one- or two-sided<br>*Only common tests should be described solely by name; describe more complex techniques in the Methods section.*
☒	☐	A description of all covariates tested
☒	☐	A description of any assumptions or corrections, such as tests of normality and adjustment for multiple comparisons
☐	☒	A full description of the statistical parameters including central tendency (e.g. means) or other basic estimates (e.g. regression coefficient) AND variation (e.g. standard deviation) or associated estimates of uncertainty (e.g. confidence intervals)
☐	☒	For null hypothesis testing, the test statistic (e.g. *F*, *t*, *r*) with confidence intervals, effect sizes, degrees of freedom and *P* value noted<br>*Give P values as exact values whenever suitable.*
☒	☐	For Bayesian analysis, information on the choice of priors and Markov chain Monte Carlo settings
☒	☐	For hierarchical and complex designs, identification of the appropriate level for tests and full reporting of outcomes
☒	☐	Estimates of effect sizes (e.g. Cohen's *d*, Pearson's *r*), indicating how they were calculated

Our web collection on statistics for biologists contains articles on many of the points above.

Software and code

Policy information about availability of computer code

Data collection	Dragonfly spinning disk confocal movies and images were acquired with Fusion 2.3.0.44 software (Andor Technology).<br>Opterra confocal movies and images were acquired with Prairie View Imaging 5.4.64.500 software (Bruker).<br>STED movies and images were acquired with Imspector 16.3.16118-w2224-win64 software (Abberior Instruments).
Data analysis	We used MATLAB 2022a software for data ploting and calculation of statistical parameters.<br>All movies and images were analyzed with Image J/Fiji.<br>The code for the theoretical model is available via GitHub at https://github.com/subhadip-physics/microtubule-bundling.git

For manuscripts utilizing custom algorithms or software that are central to the research but not yet described in published literature, software must be made available to editors and reviewers. We strongly encourage code deposition in a community repository (e.g. GitHub). See the Nature Portfolio guidelines for submitting code & software for further information.

March 2021

Data

Policy information about availability of data

All manuscripts must include a data availability statement. This statement should provide the following information, where applicable:

- Accession codes, unique identifiers, or web links for publicly available datasets
- A description of any restrictions on data availability
- For clinical datasets or third party data, please ensure that the statement adheres to our policy

Quantitative analysis datasets generated in this study are available as a Source data file. Raw image data generated during this study are available freely for non-commercial research purposes from the corresponding author on reasonable request.

Human research participants

Policy information about studies involving human research participants and Sex and Gender in Research.

Reporting on sex and gender	N/A
Population characteristics	N/A
Recruitment	N/A
Ethics oversight	N/A

Note that full information on the approval of the study protocol must also be provided in the manuscript.

Field-specific reporting

Please select the one below that is the best fit for your research. If you are not sure, read the appropriate sections before making your selection.

[x] Life sciences   [ ] Behavioural & social sciences   [ ] Ecological, evolutionary & environmental sciences

For a reference copy of the document with all sections, see nature.com/documents/nr-reporting-summary-flat.pdf

Life sciences study design

All studies must disclose on these points even when the disclosure is negative.

Sample size	Sample size was determined by looking into similar studies that performed similar types of experiments, e.g., Castrogiovanni et al., Nat Commun (2022); Trivedi et al., Nat. Commun. (2019). Sample size and the number of biological replicates for each experiment are indicated in the figure captions.
Data exclusions	When imaging spindles in a vertical orientation, the spindles that were not completely vertically oriented at all time points were excluded.
Replication	Experiments were replicated in at least 3 independent experiments.
Randomization	Within each experimental regime, cell populations were assigned randomly to a siRNA, drug or control treatment.
Blinding	Not applicable because no human subjects or animals were involved, and because additional phenotypes caused by various treatments are in most cases easily recognizable.

Reporting for specific materials, systems and methods

We require information from authors about some types of materials, experimental systems and methods used in many studies. Here, indicate whether each material, system or method listed is relevant to your study. If you are not sure if a list item applies to your research, read the appropriate section before selecting a response.

Materials & experimental systems

n/a	Involved in the study
☐	☒ Antibodies
☐	☒ Eukaryotic cell lines
☒	☐ Palaeontology and archaeology
☒	☐ Animals and other organisms
☒	☐ Clinical data
☒	☐ Dual use research of concern

Methods

n/a	Involved in the study
☒	☐ ChIP-seq
☒	☐ Flow cytometry
☒	☐ MRI-based neuroimaging

Antibodies

Antibodies used

For immunofluorescence:
Primary antibodies
- Mouse monoclonal PRC1 (sc-376983, Santa Cruz Biotechnology), diluted 1:100
- Rat anti-alpha-tubulin YL1/2 (MA1-80017, Invitrogen, CA, SAD), diluted 1:500
- Mouse IgG monoclonal anti-GFP (Ref 11814460001, LOT42903200, Roche), diluted 1:100
- Rabbit anti-alpha-tubulin (SAB4500087, LOT 310379, Sigma Aldrich), diluted 1:500
- Human anti-CREST (15-235, Antibodic sinc), 1:100
- Rabbit anti-CENP-E (ab133583, Abcam, Cambridge, UK), diluted 1:500
- Rabbit anti-Aurora B (ab239837, Abcam), diluted 1:100
- Mouse anti-Hec1 (ab3613, Abcam), diluted 1:100
- Rabbit anti-Spindly (A301-354A, Biomolecules), diluted 1:100
-Rabbit anti-phospho-CENP-A (Ser7) (07-232, Sigma-Aldrich), diluted 1:500
Secondary antibodies
- Donkey anti-mouse IgG Alexa Fluor 594 (ab150112, Abcam, Cambridge, UK), diluted 1:250
- Donkey anti-mouse IgG Alexa Fluor 647 (A31571, Invitrogen), diluted 1:1000
- Donkey anti-rabbit IgG Alexa Fluor 647 (ab150075, Abcam), diluted 1:500
- Donkey anti-rat IgG Alexa Fluor 647 (ab150155, Abcam), diluted 1:500
- Goat anti-human IgG 594 (ab96909, Abcam), diluted 1:500
For immunoblotting:
Primary antibodies
- Mouse anti-CENP-E (sc-376685, Santa Cruz Biotechnologies), diluted 1:500
- Mouse anti-vinculin (SAB4200729-100UL, Sigma Aldrich), diluted 1:5000
Secondary antibodies
- HRP-conjugated secondary antibodies (Jackson ImmunoResearch), diluted 1:10000

Validation

All used antibodies are commercially available and validated before.
Primary antibodies :
- Mouse monoclonal PRC1 (sc-376983, Santa Cruz Biotechnology) is validated for IF by the manufacturer and cited in 17 refs as reported on the manufacturer website (https://www.scbt.com/p/prc1-antibody-c-1), Species Reactivity : mouse, rat, human
- Rat anti-alpha-tubulin YL1/2 (MA1-80017, Invitrogen, CA, SAD) is validated for IF by the manufacturer and cited in 42 refs as reported on the manufacturer website (https://www.thermofisher.com/antibody/product/alpha-Tubulin-Antibody-clone-YL1-2-Monoclonal/MA1-80017), Species Reactivity : Dog, Fruit fly, Human, Mouse, Plant, Pig, Rabbit, Rat, Xenopus, Yeast
- Mouse IgG monoclonal anti-GFP (Ref 11814460001, LOT42903200, Roche), validated for IF by the manufacturer and cited in 623 refs as reported on the manufacturer website (https://www.sigmaaldrich.com/HR/en/product/roche/11814460001)
- Rabbit anti-alpha-tubulin (SAB4500087, LOT 310379, Sigma Aldrich) is validated for IF by the manufacturer and cited in 12 refs as reported on the manufacturer website (https://www.sigmaaldrich.com/HR/en/product/sigma/sab4500087), Species Reactivity : human, mouse, rat
- Human anti-CREST (15-235, Antibodic sinc) is validated for IF by the manufacturer as reported on the manufacturer website (https://www.antibodiesinc.com/products/anti-centromere-protein-antibody-fitc-labeled-15-235-f), Species Reactivity : Hamster, Human, Mouse, Rat
- Rabbit anti-CENP-E (ab133583, Abcam, Cambridge, UK) is validated for IF by the manufacturer and cited in 6 refs as reported on the manufacturer website (https://www.abcam.com/cenpe-antibody-epr45422-ab133583.html), Species Reactivity : human
- Rabbit anti-Aurora B (ab239837, Abcam) is validated for IF by the manufacturer as reported on the manufacturer website (https://www.abcam.com/aurora-b-antibody-ep1009y-bsa-and-azide-free-ab239837.html), Species Reactivity : human
- Mouse anti-Hec1 (ab3613, Abcam) is validated for IF by the manufacturer and cited in 102 refs as reported on the manufacturer website (https://www.abcam.com/hec1hec-antibody-9g3-ab3613.html) Species Reactivity : human, pig
- Rabbit anti-Spindly (A301-354A, Biomolecules) is validated for IF as described in Etemad et al., J Cell Sci., 2019, Species Reactivity : human
-Rabbit anti-phospho-CENP-A (Ser7) (07-232, Sigma-Aldrich) is validated for IF as described in Eot-Houllier et al., Nat. Commun., 2018, Species Reactivity : human
- Mouse anti-CENP-E (sc-376685, Santa Cruz Biotechnology) is validated for WB by the manufacturer and cited in 5 refs as reported on the manufacturer website (https://www.scbt.com/p/cenp-e-antibody-c-5)
- Mouse anti-Vinculin (SAB4200729-100UL, Sigma-Aldrich) is validated for WB by the manufacturer and cited in 23 refs as reported on the manufacturer website (https://www.sigmaaldrich.com/HR/en/product/sigma/sab4200729)

Secondary antibodies:
- Donkey anti-mouse IgG Alexa Fluor 594 (ab150112, Abcam, Cambridge, UK) is validated for IF by the manufacturer and cited in 10 refs as reported on the manufacturer website (https://www.abcam.com/donkey-mouse-igg-hl-alexa-fluor-594-preadsorbed-ab150112.html)
- Donkey anti-mouse IgG Alexa Fluor 647 (A31571, Invitrogen) is validated for IF by the manufacturer and cited in 1410 refs as reported on the manufacturer website (https://www.thermofisher.com/antibody/product/Donkey-anti-Mouse-IgG-H-L-Highly-Cross-Adsorbed-Secondary-Antibody-Polyclonal/A-31571), Species Reactivity : mouse
- Donkey anti-rabbit IgG Alexa Fluor 647 (ab150075, Abcam) is validated for IF by the manufacturer and cited in 237 refs as reported on the manufacturer website (https://www.abcam.com/donkey-rabbit-igg-hl-alexa-fluor-647-ab150075.html),
- Donkey anti-rat IgG Alexa Fluor 647 (ab150155, Abcam) is validated for IF by the manufacturer and cited in 51 refs as reported on the manufacturer website (https://www.abcam.com/donkey-rat-igg-hl-alexa-fluor-647-preadsorbed-ab150155.html)
- Goat anti-human IgG 594 (ab96909, Abcam) is validated for IF by the manufacturer as reported on the manufacturer website (https://www.abcam.com/goat-human-igg-hl-dylight-594-ab96909.html)

Eukaryotic cell lines

Policy information about cell lines and Sex and Gender in Research

Cell line source(s)	- Unlabeled human HeLa-TDS cells from the High-Throughput Technology Development Studio (Max Planck Institute of Molecular Cell Biology and Genetics, Dresden, Germany),<br>- HeLa-Kyoto BAC lines stably expressing PRC1-GFPare courtesy of Ina Poser and Tony Hyman (Max Planck Institute of Molecular Cell Biology and Genetics, Dresden, Germany),<br>- Human hTERT-RPE1cells stably expressing CENP-A-GFP and Centrin1-GFP, courtesy of Alexey Khodjakov (Wadsworth Center, New York State Department of Health, Albany, NY, USA)
Authentication	None of the cell lines was authenticated by the authors.
Mycoplasma contamination	Cells were tested regularly for Mycoplasma contamination using DAPI and were found to be negative for Mycoplasma contamination.
Commonly misidentified lines (See ICLAC register)	No commonly misidentified lines were used in this study.
